# Brain is a potential sanctuary for subtype C HIV-1 irrespective of ART treatment outcome

**DOI:** 10.1371/journal.pone.0201325

**Published:** 2018-07-24

**Authors:** For Yue Tso, Guobin Kang, Eun Hee Kwon, Peter Julius, Qingsheng Li, John T. West, Charles Wood

**Affiliations:** 1 Nebraska Center for Virology and the School of Biological Sciences, University of Nebraska-Lincoln, Nebraska, United States of America; 2 Department of Pathology and Microbiology, University Teaching Hospital, Nationalist Road, Lusaka, Zambia; University of Pittsburgh, UNITED STATES

## Abstract

Subtype C HIV-1 is responsible for the largest proportion of people living with HIV-1 infection. However, there is limited information about the roles of the brain and its cell types as a potential sanctuary for this subtype and how the sanctuary may be affected by the administration of anti-retroviral therapy (ART). To address this issue, we collected postmortem brain tissues from ART treated HIV-1 infected Zambian individuals who experienced complete viral suppression and those who did not. Tissues from various brain compartments were collected from each individual as frozen and formalin-fixed paraffin embedded brain specimens, for detection and quantification of HIV-1 genomes and identification of the infected cell type. Genomic DNA and RNA were extracted from frozen brain tissues. The extracted DNA and RNA were then subjected to droplet digital PCR for HIV-1 quantification. RNA/DNAscope in situ hybridization (ISH) for HIV-1 was performed on formalin-fixed paraffin embedded brain tissues in conjugation with immunohistochemistry to identify the infected cell types. Droplet digital PCR revealed that HIV-1 *gag* DNA and RNA were detectable in half of the cases studied regardless of ART success or failure. The presence of HIV-1 lacked specific tissue compartmentalization since detection was random among various brain tissues. When combined with immunohistochemistry, RNA/DNAscope ISH demonstrated co-localization of HIV-1 DNA with CD68 expressing cells indicative of microglia or peripheral macrophage. Our study showed that brain is a potential sanctuary for subtype C HIV-1, as HIV-1 can be detected in the brain of infected individuals irrespective of ART treatment outcome and no compartmentalization of HIV-1 to specific brain compartments was evident.

## Introduction

The introduction of combined anti-retroviral therapy (ART) has been an important milestone in curtaining the HIV-1 AIDS epidemic and has led to drastic improvement in the prognosis of HIV-1 infected individuals. Although the ART regimens have extended the life expectancy of many individuals, prolonged treatment comes with an increased probability of adverse effects [1]. Emergence of ART resistant HIV-1 variants and other non-AIDS associated complications such as HIV-1 associated neurocognitive disorders (HAND) and cancers continue to be problematic despite the effective use of ART [2]. Additionally, the high cost of ART has placed a heavy financial burden on resource-limited countries, such as those in sub-Saharan Africa, and such burden potentially limit the universal access and sustainability of ART implementation in these countries [3].

The ability of HIV-1 to persist in the infected host under suppressive ART has proven to be a formidable obstacle to the eradication of HIV-1. Despite its capacity to reduce productive infectious HIV-1 in the peripheral circulation to undetectable levels, ART does not eradicate latent HIV-1, and therefore treatment has to be lifelong without discontinuation. This may prove financially difficult to sustain in perpetuity. Hence, there has been an emphasis on the development of latency-reversing agents (LRAs) with the goal to reactivate latent HIV-1, followed by therapeutic intervention in the “shock and kill” strategy [4]. However, for such latency reversal strategies to be most effective, it requires a better understanding of where latent HIV-1 sanctuaries exist in infected individuals, whether those sanctuaries are modulated by treatment outcomes and vary with different HIV-1 subtypes.

The central nervous system (CNS) has been documented as a potential sanctuary for HIV-1 and the virus can readily be detected in the cerebrospinal fluid (CSF) early after HIV-1 infection [5, 6]. The CNS is protected by the blood-brain barrier (BBB) and is considered an immunological and pharmacological privileged site to which ART has limited access [7–10]. Thus, the CNS presents a favorable environment for HIV-1 persistence. The presence of HIV-1 in the CNS has been associated with increased risk of developing HAND in HIV-1 infected individuals who are ART naïve or have experienced therapy failure [11]. Additionally, a substantial proportion of ~18% HIV-1 infected individuals achieving viral suppression also experienced neuropsychological impairment [12].

HIV-1 infection of the CNS has primarily been studied in subtype B HIV-1 infected individuals [5, 13, 14]. Despite the fact that subtype C HIV-1 is responsible for the largest proportion of people living with HIV-1 infection, there is limited information about the roles of the brain as a potential sanctuary for this subtype and how the sanctuary may be affected by the administration of ART [15, 16]. The inaccessibility of brain tissues from HIV-1 infected individuals makes sampling difficult and any longitudinal study impossible. In this study, we were able to collect postmortem brain tissues from ART treated, subtype C HIV-1 infected, individuals from Zambia. We performed a cross-sectional examination for the presence of HIV-1 in the brain tissues of those who were virally suppressed versus those who had experienced ART failures. We utilized ultra-sensitive detection methods such as droplet digital-PCR (ddPCR) and RNA/DNAscope in situ hybridization (ISH) in conjugation with traditional immunohistochemistry (IHC) to detect low HIV-1 copy in the frozen and formalin-fixed paraffin embedded brain tissues respectively [17–19]. Our study is significant as it addresses important knowledge gaps as to whether the CNS can serve as a potential sanctuary for subtype C HIV-1 in infected individuals and how the sanctuary is impacted by ART treatment.

## Materials and methods

### Postmortem brain sample

Permission to conduct this study was obtained from the University of Zambia Biomedical Research Ethics Committee and the Institutional Review Board of the University of Nebraska-Lincoln. Upon notification of a death in the adult medical wards at the University Teaching Hospital (UTH) in Zambia, family members of the deceased were approached by an experienced team member. Grief counseling was offered to the family members present regardless of their decision to consent or not. The goals of the study were then explained as well as the procedures to be conducted for tissue collection during autopsy. For those that provided written consent, postmortem samples were collected at 24 hours after death from the following brain compartments: frontal lobe, cerebellum, hippocampus, basal ganglia, temporal lobe, parietal lobe and occipital lobe. Representative tissue slice (at least 3 mm) for each brain compartment was dissected and divided for snap freezing in liquid nitrogen and fixation in 4% paraformaldehyde for paraffin-embedding. Venous blood was also collected if possible.

### RT-PCR for plasma HIV-1 RNA load

HIV-1 RNA was extracted from plasma with QIAamp Viral RNA Mini Kit (Qiagen, Hilden, Germany) according to the manufacturer’s protocol with on-column DNase I treatment. HIV-1 plasma viral load was quantified with RNA UltraSense One-Step Quantitative RT-PCR System (Invitrogen, Waltham, MA) using the following conditions: 50°C for 15 min; 95°C for 2 min; 40 cycles at 95°C for 15 sec and 60°C for 1 min. The primers and probe target the HIV-1 *gag* as previously described [17]. AcroMetrix HIV-1 panel (ThermoFisher Scientific, Fremont, CA) was used for standard curve.

### Extraction of genomic DNA and RNA from postmortem brain tissues

250 to 300 mg of each fresh-frozen brain tissue were pulverized by cryo-cracking in liquid nitrogen. Cryo-cracked tissues were incubated overnight at 55°C with cell lysis solution and proteinase K (Qiagen, Hilden, Germany). The sample was then chilled on ice for 5 mins before the addition of protein precipitation solution, mix and spun twice at 2000 x *g* for 10 mins at 4°C to pellet the protein precipitates. The supernatant was transferred to a new tube with glycogen and isopropanol to precipitate both genomic DNA and RNA simultaneously. The mixture was then spun at 2000 x *g* for 5 mins at 4°C to pellet the genomic DNA and RNA. The DNA/RNA pellet was washed twice with 70% ethanol, air-dried, resuspended in hydration solution (Qiagen, Hilden, Germany) and incubated at 65°C for 30 mins to dissolve the pellet.

To separate the genomic RNA from DNA, Ambion TRIzol LS reagent (Invitrogen, Waltham, MA) was added to the genomic DNA/RNA solution according to the manufacturer’s protocol. After phase separation, genomic RNA from the aqueous layer was extracted using QIAgen miRNeasy mini kit (Qiagen, Hilden, Germany) according to manufacturer’s protocol with additional on-column DNase I treatment.

To extract the genomic DNA, the aqueous layer after phase separation was mixed with glycogen and 100% ethanol for 5 mins before spun at 15000 x *g* for 5 mins at 4°C. After the supernatant was discarded, the genomic DNA pellet was washed twice with 0.1M sodium citrate in 10% ethanol for 30 mins at room temperature and spun at 15000 x *g* for 5 mins at 4°C. The genomic DNA pellet was then washed with 75% ethanol for 10 mins at room temperature, spun at 15000 x *g* for 5 mins at 4°C. The genomic DNA pellet was air-dried, resuspended in hydration solution (Qiagen, Hilden, Germany) and incubated at 65°C for 1 hr before overnight incubation at room temperature to ensure the DNA pellet was completely solubilized. The genomic DNA was then further purified through standard ethanol precipitation.

Concentration of the extracted genomic DNA and RNA was determined using Qubit double-stranded DNA and RNA broad-range kits (Invitrogen, Waltham, MA), and measured by Qubit fluorometer (Invitrogen, Waltham, MA).

### ddPCR for HIV-1 DNA and RNA

HIV-1 copy numbers from the extracted genomic DNA and RNA were determined by ddPCR, as previously described [17]. Briefly, for detection of HIV-1 DNA, the extracted genomic DNA was first digested with restriction enzyme *Msc* I to reduce sample viscosity and increase template accessibility. The target sequences does not contain *Msc* I recognition sites. Each DNA ddPCR reaction consisted of 1X ddPCR supermix for probes (Biorad, Hercules, CA), 900 nM each of HIV-1 *gag* forward and reverse primers, 250 nM FAM-labeled HIV-1 *gag* probe, template DNA and top up to 20 μl with molecular grade water [17]. The reaction mixture was then loaded into QX100 droplet generator (Biorad, Hercules, CA) for droplet emulsion generation and PCR was performed with C1000 Touch Thermal Cycler (Biorad, Hercules, CA). Fluorescence signal was quantified and analyzed by the QX100 droplet reader (Biorad, Hercules, CA) and QuantaSoft version 1.3.2.0 (Biorad, Hercules, CA) respectively. To determine the total number of analyzed cells, a separate ddPCR reactions were performed against the beta-globin gene. The cut-off value for ddPCR positivity was determined using the genomic DNA extracted from a total of 6.74 x 10^7^ cells from various brain tissues of an HIV-1 negative individual (44 years old, male, cause of death as cardiac arrest), and was consistently found to be at an average of ~1 HIV-1 copy/10^6^ cells. Brain tissue from a HIV-1 positive (30 years old, male, cause of death as toxoplasmosis) ART naïve individual was used as positive control.

Detection of HIV-1 RNA was similar to the DNA method, except that the reaction mix consisted of 1X One-Step RT-ddPCR supermix (Biorad, Hercules, CA) and an additional 1 mM of manganese acetate solution. A total of 120 ng of genomic RNA from each brain compartment was analyzed for HIV-1 *gag* RNA. The cut-off value for ddPCR positivity was determined using genomic RNA extracted from the various brain tissues of an HIV-1 negative individual, and signals above ~2 HIV-1 copies/μg of RNA input were determined to be true signals. The mean viral copies and statistical analysis (Unpaired T test) were performed using GraphPad Prism 5 (GraphPad Software, La Jolla, CA).

### RNA/DNAscope ISH and IHC

HIV-1 DNA and RNA in brain tissues were detected using DNAscope ISH sense and antisense riboprobes in combination with RNAscope 2.0 HD red reagent kit (Advanced Cell Diagnostics, CA), respectively. The experiment was conducted according to kit’s instruction and previously reported protocol [20]. The ISH stained sections were then digitized using Aperio CS2 Scanscope (Leica Aperio, CA). For determining the cell types of HIV-1 RNA and DNA positive cells, the coverslip of viral RNA or viral DNA positive tissue sections were removed and tissue sections were rehydrated and received antigen retrieval as previously reported [21]. CD68^+^ cells or CD4^+^ T cells were immunohistochemically stained with mouse anti-human CD68 monoclonal antibody (KP1, 1:200, Abcam) or rabbit anti-human CD4 monoclonal antibody (EPR6855, 1:200, Abcam) and the signal was developed with the Dako Envision and Peroxidase kit using diaminobenzidine (DAB) as substrate. Stained sections were digitized and CD68^+^ cells or CD4^+^ T cells were analyzed by Aperio’s Spectrum Plus analysis program (version 9.1; Aperio ePathology Solutions) as described previously [22].

## Results

A total of 8 HIV-1 positive individuals were studied. These subjects had been on ART for >6 months (range 9–108 months) prior to death (Table 1). The ART regimens of these subjects were similar, consisting primarily of a protease inhibitor, nucleoside reverse transcriptase inhibitors and non- nucleoside reverse transcriptase inhibitors. Despite the administration of ART over extended period of time, half of the cohort were considered to be ART failures given the detectable plasma HIV-1 RNA ranging from 4 x 10^2^ to 1 x 10^5^ copies/ml (Table 1) despite treatment for >6 months. The remainder were virally suppressed with undetectable plasma HIV-1 RNA by RT-PCR. There was no correlation between the duration of ART and the outcome of ART therapy. The frontal lobe, cerebellum, hippocampus, basal ganglia, temporal lobe, parietal lobe and occipital lobe of the brain from each subject were used for subsequent analyses.

**Table 1:** Subject demographic information.

Subject	Age	Sex	Last known CD4 count (cells/μl)	Plasma HIV-1 RNA load (copies/ml)	ART duration (months)	ART regiment	Clinical diagnosis
**245**	40	Male	Undetermined	Undetectable	48	Tenofovir, Emtricitabine, Efavirenz	Cor pulmonale with cardiogenic shock
**328**	37	Male	Undetermined	Undetectable	10	Tenofovir, Emtricitabine, Efavirenz	Meningo-encephalitis
**332**	37	Female	Undetermined	Undetectable	24	Tenofovir, Emtricitabine, Efavirenz	Retroviral disease, upper gastrointestinal tract bleeding, gastric ulcer
**408**	17	Male	900	Undetectable	108	Tenofovir, Emtricitabine, Nevirapine	Retroviral disease, chronic meningitis
**257**	40	Male	Undetermined	3 x 10^4^	9	Tenofovir, Emtricitabine, Efavirenz	Retroviral disease, anaemia
**283**	34	Female	300	4 x 10^2^	36	Tenofovir, Emtricitabine, Nevirapine	Retroviral disease, tuberculosis
**309**	34	Female	318	2 x 10^4^	96	Tenofovir, Emtricitabine, Efavirenz	Retroviral disease, cerebrovascular accident
**319**	44	Female	70	1 x 10^5^	24	Lopinavir, Abacavir, Lamivudine	Retroviral disease, anaemia

**Fig 1:**
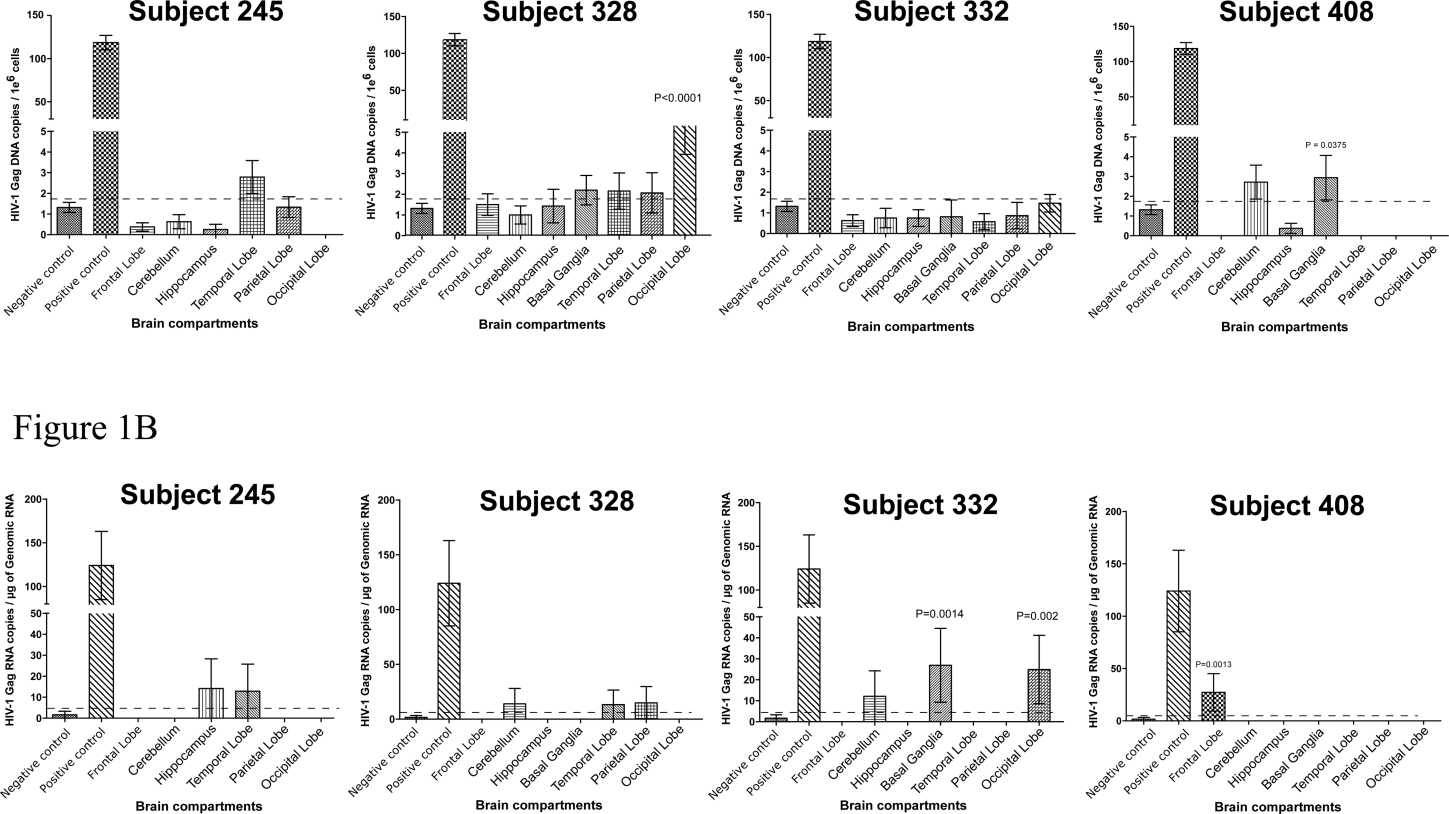
Droplet digital PCR detection of HIV-1 DNA and RNA in brain tissues of viral suppressed individuals. (A) HIV-1 *gag* DNA copies per million cells in various brain tissues (B) HIV-1 *gag* RNA copies per million cells in various brain tissues. Basal Ganglia was not available for subject 245. P-value indicate statistically significant from background/negative control. Dash lines indicate cut-off value for ddPCR positivity based on the negative control. Negative control derived from brain tissue of a HIV-1 negative individual. Positive control derived from brain tissue of a HIV-1 positive but ART naïve individual.

Among the virally suppressed aviremic subjects, three out of four cases have detectable viral DNA or RNA or both. One case (subject 245) has no statistical significant copies of either viral DNA or RNA (Fig 1A and 1B). For subject 408, both viral DNA and RNA were detected in some tissues. Low but statistical significance copies of HIV-1 *gag* DNA, at 3 copies/10^6^ cells (P = 0.0375) were detected in the basal ganglia (Fig 1A). The frontal lobe from this subject also contained HIV-1 *gag* RNA at 27 copies/μg of genomic RNA input (P = 0.0013) (Fig 1B). For subject 332 statistically significant HIV-1 *gag* RNA level was distinctly detected in the basal ganglia and occipital lobe at 26 copies/μg of genomic RNA input (P = 0.0014) and 25 copies/μg of genomic RNA input (P = 0.002), respectively (Fig 1B). However, even though some viral DNA was only detected in occipital lobe but the copy number is too low to be significant. Interestingly, for subject 328, low but consistent and statistically significant copies of viral DNA were detected by ddPCR in the occipital lobe at 5 copies/10^6^ cells (P < 0.0001). No significant copy number of viral RNA was detected in any tissue tested for this subject.

**Fig 2:**
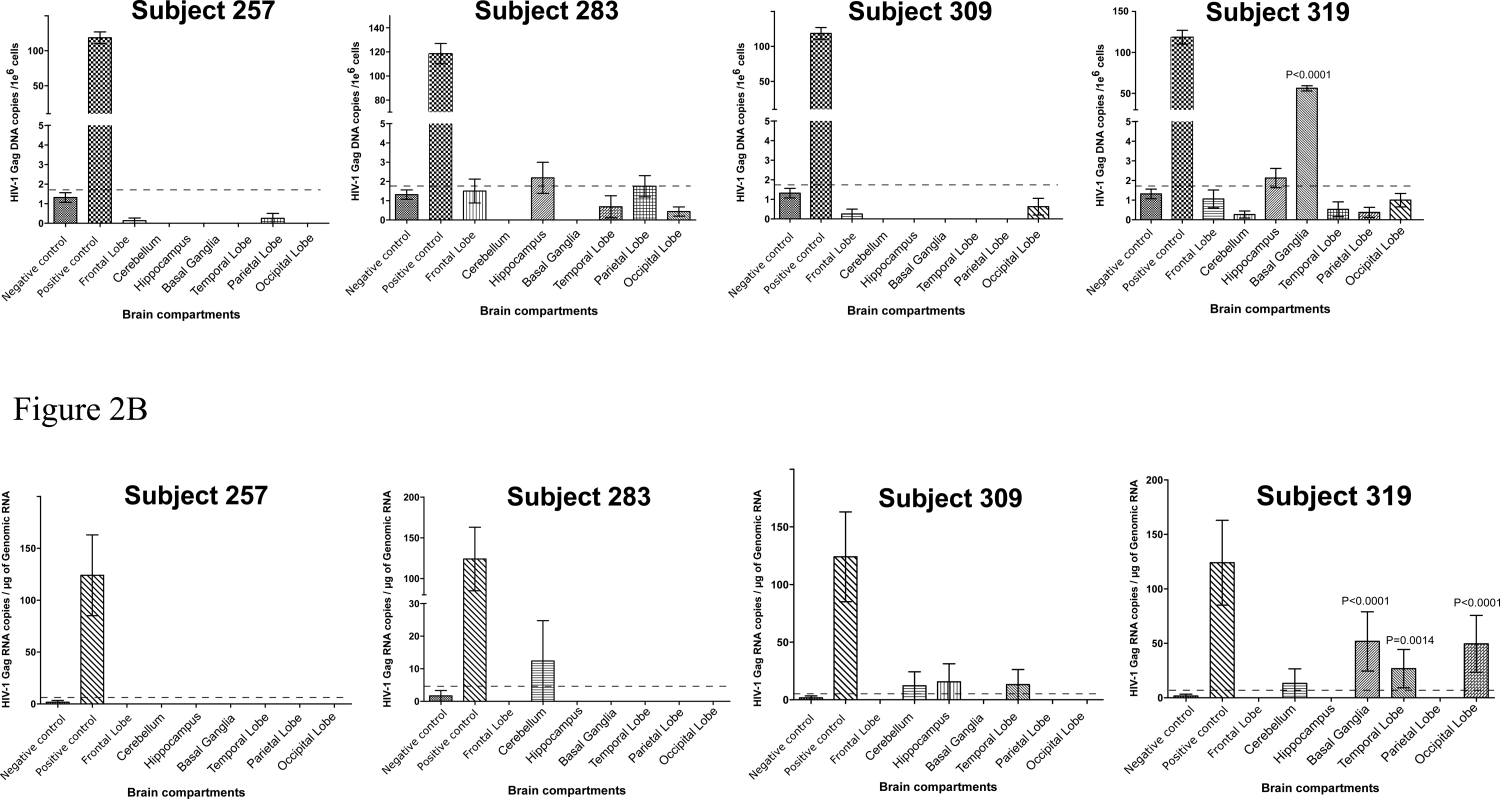
Droplet digital PCR detection of HIV-1 DNA and RNA in brain tissues of ART failure individuals. (A) HIV-1 *gag* DNA copies per million cells in various brain tissues (B) HIV-1 *gag* RNA copies per million cells in various brain tissues. P-value indicate statistically significant from background/negative control. Dash lines indicate cut-off value for ddPCR positivity based on the negative control. Negative control derived from brain tissue of a HIV-1 negative individual. Positive control derived from brain tissue of a HIV-1 positive but ART naïve individual.

Surprisingly, among the individuals failing to control viral load while on ART, only one of four cases has detectable viral DNA and RNA. HIV-1 *gag* DNA that was statistically distinct from the background was only detected in the basal ganglia of subject 319 that had the highest level of HIV-1 *gag* DNA in the entire cohort, at 56 copies/10^6^ cells (P < 0.0001) (Fig 2A). This subject also had significant amount of HIV-1 *gag* RNA in the basal ganglia, temporal lobe and occipital lobe at 52, 27 and 50 copies/μg of input genomic RNA respectively (Fig 2B). No significant *gag* RNA was detected among the three ART failure individuals in their tested brain tissues.

**Fig 3:**
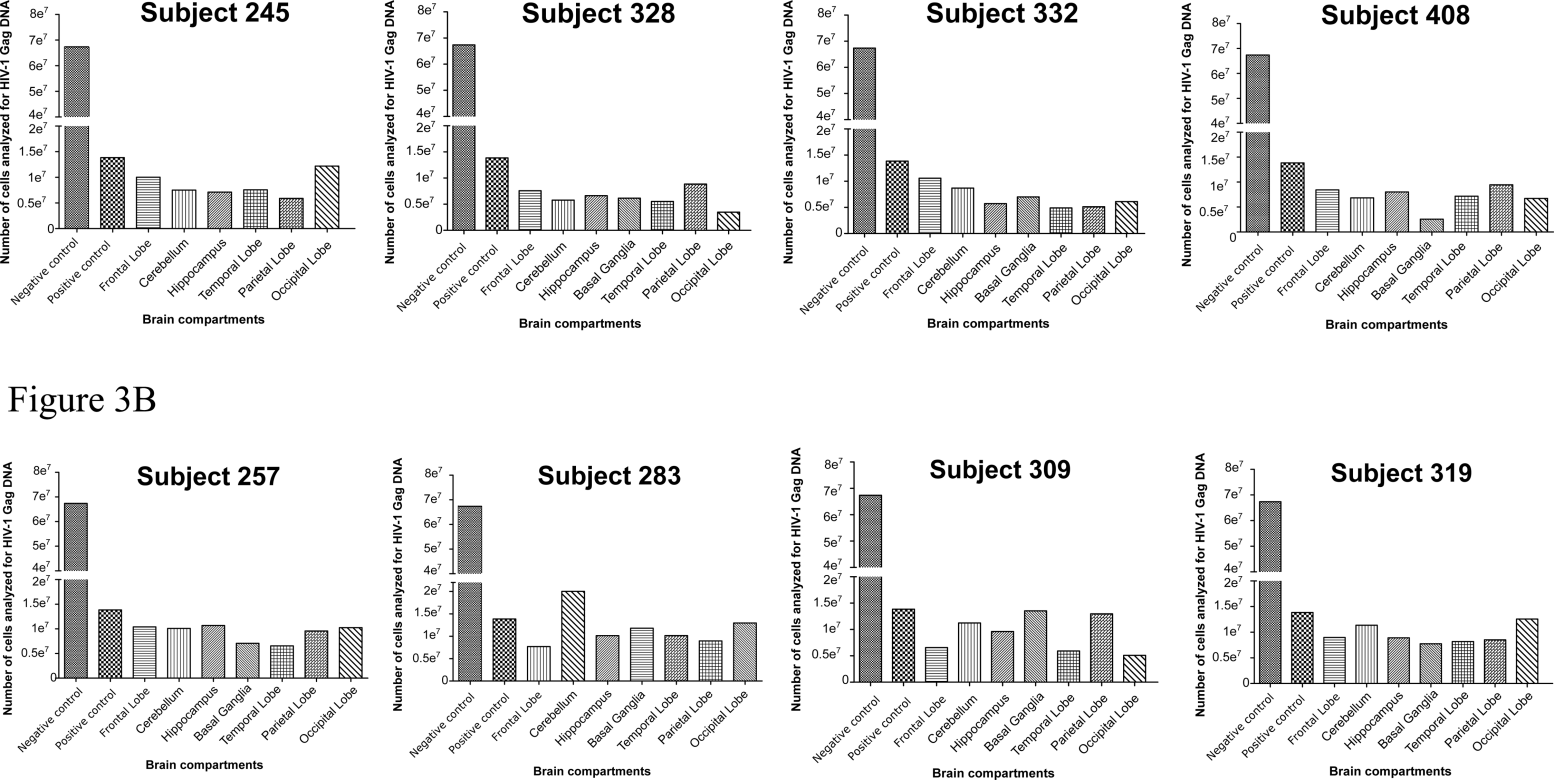
Number of cells analyzed by droplet digital PCR for HIV-1 DNA and RNA in brain tissues. (A) Viral suppressed subjects (B) ART failure subjects.

**Fig 4:**
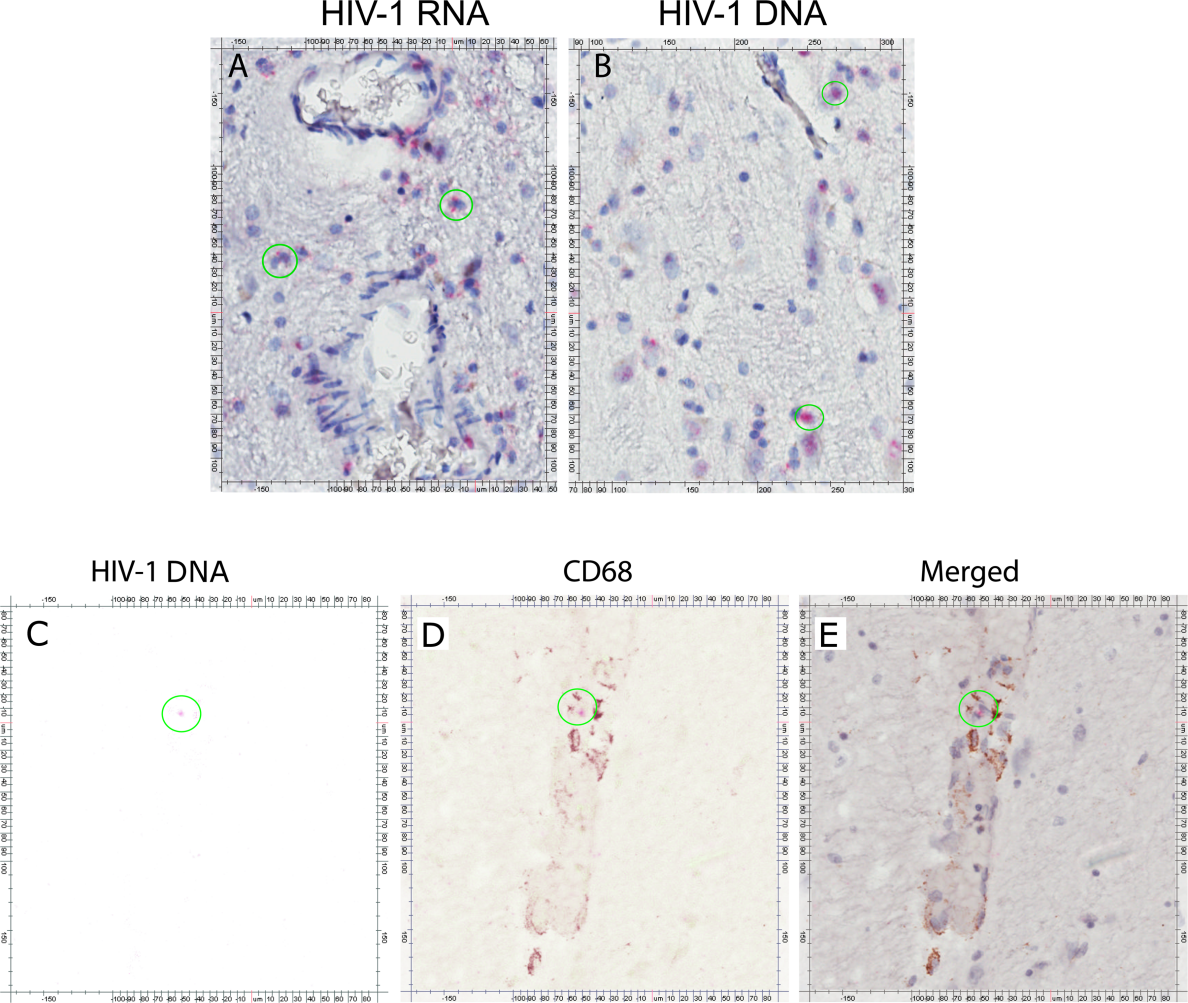
RNA/DNAscope in situ hybridization for HIV-1 RNA and DNA detection. (A) HIV-1 RNA in basal ganglia of subject 319. (B) HIV-1 DNA in basal ganglia of subject 319. (C) HIV-1 DNA in basal ganglia of subject 408. (D) CD68 expression in basal ganglia of subject 408 (E) Merged of HIV-1 DNA and CD68 expression in basal ganglia of subject 408. HIV-1 RNA/DNA positive signals are shown as red color. Nucleus are shown as blue color. CD68 expression are shown as brown color. Green circles highlight representative cells positive for HIV-1 RNA or DNA and/or CD68 expression.

The lack of detection for both the HIV-1 *gag* DNA and RNA in some of the brain samples was not likely due to insufficient number of cells analyzed. On average, about 5 x 10^6^ cellular equivalents were used for ddPCR analysis for both the virally suppressed and ART failure cases (Fig 3A and 3B respectively). Furthermore, the basal ganglia from subject 408 had the least number of cells analyzed with only 2.6 x 10^6^ cells (Fig 3A), but the HIV-1 *gag* DNA was still detectable. The ddPCR detection of HIV-1 DNA and RNA from the brain tissues was confirmed with RNA/DNAscope ISH on selected tissues. For example, the presence of HIV-1 RNA and DNA were evident in the basal ganglia of ART failure subject 319, where both viral RNA and DNA expressing cells were detected (Fig 4A and 4B, respectively). Likewise, RNA/DNAscope ISH results from a viral suppressed subject 408, whose basal ganglia had ddPCR detectable viral DNA, but not viral RNA, showed only viral DNA positive cells (Fig 4C).

We then proceeded to determine the identity of the viral DNA positive infected cells in the basal ganglia of subject 408 by performing IHC for CD4^+^ T cells or CD68^+^ expressing cells that are indicative of microglia or peripheral macrophage, in conjunction with DNAscope ISH against HIV-1 DNA. Although we only detected very few CD4^+^ T cells in the brain tissues (data not shown), cells expressing CD68^+^ were readily detected in the brain tissues and, more importantly, overlapped with HIV-1 DNA signal from DNAscope ISH (Fig 4C, 4D and 4E). This result suggests that microglia or peripheral macrophage that infiltrated the brain are the major cell types infected by subtype C HIV-1 in the brain, at least based on the cases that we have analyzed.

## Discussion

The existence of a brain sanctuary in HIV-1 infected individuals on suppressive ART is still controversial and has not been studied extensively due to difficulties in obtaining tissues for such analysis. In addition, most studies on HIV-1 infection of the central nervous system to date have focused on subtype B strains [23, 24]. A previous study reported subtype C HIV-1 compartmentalization in the central nervous system in HIV-1 infected children [25]. However, that study only examined the cerebrospinal fluid without access to parenchymal brain tissues. Although another study had explored the presence of subtype C HIV-1 in brain tissues from autopsy, it only examined proviral DNA in ART naïve cases [26]. It had no ART treated cases for comparison and did not address the question of viral reactivation. Our postmortem study design directly addresses the question of whether the brain can serve as a sanctuary for subtype C HIV-1 by examining parenchymal tissues from sub-Saharan African ART treated individuals. Subtype C HIV-1 is the dominant strain in Zambia and the subtype C classification for our sample was also confirmed by sequencing for the viral envelope from subject 328 [27]. Our findings are particularly relevant since subtype C HIV-1 is the most prevalent strain present in >50% of individuals living with HIV-1 worldwide.

HIV-1 infection is known to cause chronic inflammation, which could lead to dysregulation of the tight junction between the brain microvascular endothelial cells and permeabilization of the blood-brain barrier, thereby potentially increase HIV-1 infiltration into the brain [28–30]. Additionally, the failure of ART to suppress the HIV-1 viral load in the peripheral blood would have been predicted to increase the chances for HIV-1 to infect the CNS. Several groups have previously reported that subtype B HIV-1 can be detected from the brain tissues in about 50–55% of HIV-1 infected and ART treated patients [23, 31]. Similar to these reports, our data also showed a 50% HIV-1 detection rate, with 4 out of the 8 subtype C HIV-1 infected and ART treated subjects having detectable HIV-1 DNA or RNA in brain tissues. Despite the lack of HIV-1 in most cases, we cannot rule out the presence of virus in these ‘negative’ samples since we can only analyzed a small section of each brain tissue, or the virus could be present at a frequency below our detection limit. Nevertheless the detection of viral DNA in the absence of viral RNA is of important even though we cannot determine whether the provial DNA detected represent defective or intact viral genome. Surprisingly, our data did not show differential HIV-1 infection frequency in the brains of ART failure cases compared to those with fully suppressed peripheral viral load. One possible explanation is that, in addition to our analyses were from a limited section of the brain tissues, subtype C HIV-1 might be less neurotropic than subtype B. This notion may be supported by previous reports suggesting that subtype C HIV-1 causes less pathological changes in the brain than subtype B in both human patients and experimentally infected animals [32, 33].

Previous studies have also suggested that certain brain compartments, such as the basal ganglia, temporal lobe and hippocampus, have an increased tendency to harbor HIV-1 [34, 35]. Although HIV-1 DNA or RNA was detected in the basal ganglia and temporal lobe from several individuals in our study, we did not observe any obvious pattern in the distribution of detectable events among the various brain compartments. Since our data revealed no specific brain compartment as being more susceptible to subtype C HIV-1 infection than others, we conclude that infection of the various brain compartments likely results from random events.

The distribution of detectable HIV-1 RNA was also not compartment-specific and moreover did not correlate with the outcome of ART. Since we employed a primer set that targeted the HIV-1 *gag* gene, which is typically encoded by genome length unspliced transcripts, we eliminated the possibility of detecting prematurely terminated short viral transcripts derived solely from the long-terminal repeat [36]. Detection of HIV-1 *gag* RNA in brain tissue, strongly suggests that there could be persistent low-level viral replication or reactivation in the brain tissues despite administration of ART. A recent non-human primate study with simian immunodeficiency virus followed by comparative phylogenetic analysis of the viral sequences in the cerebrospinal fluid versus plasma also suggested that HIV-1 can indeed be reactivated in the brain [37, 38]. In contrast, some of our samples had detectable HIV-1 *gag* RNA but no viral DNA. The detected virus, presumably in the vasculature, might therefore have originated from an unsampled region within the same tissue, from other brain compartments, or even from another peripheral tissue reservoir. It is also possible that viral DNA was present but simply below our detection limit, or we have missed the sections that were positive.

The precise mechanism that HIV-1 utilizes to enter the brain has not been absolutely defined, but is thought to involve both infected T-cells and cell-free virions infiltrating past the blood-brain barrier. Once in the brain parenchyma, HIV-1 cannot infect neurons, but is known to infect CD68^+^ microglia [39, 40]. To identify the infected cell type in our cohort, we performed IHC in conjunction with DNAscope ISH for HIV-1 DNA on selected brain tissues. Our data showed that the HIV-1 DNA detected in the brain resides within CD68 expressing microglia cells or brain-infiltrating peripheral macrophages. However, there are several limitations to our present study. First is the small number of cases studied mainly due to the difficulties in obtaining these postmortem cases especially in the African setting, a future study with a much larger cohort will be needed to support our preliminary findings. Furthermore, at this juncture, it is not possible to determine whether the viral genomes detected are from intact and therefore reactivable viral genomes or not since viral outgrowth assays have not yet been successfully performed with postmortem brain tissues. Nevertheless, the presence of HIV-1 DNA in the absence of viral RNA at least in one of our cases suggests the possibility of functional latent sanctuary in the brain that could repopulate the periphery upon treatment cessation.

In conclusion, our study has shown that brain from subtype C HIV-1 infected individuals can harbor viral genomes and can be a potential sanctuary, regardless of the peripheral success or failure of ART. The virus appears to be randomly distributed among the various brain compartments with evidence of low copies of viral RNA in some cases, indicative of ongoing persistent viral replication. Such a sanctuary may present a major challenge for HIV-1 treatments as the capacity to penetrate the blood-brain barrier will need to be taken into consideration.
